# Quick-SOFA score to predict mortality among geriatric patients with influenza in the emergency department

**DOI:** 10.1097/MD.0000000000015966

**Published:** 2019-06-07

**Authors:** Su-Hen Chang, Chien-Chun Yeh, Yen-An Chen, Chien-Chin Hsu, Jiann-Hwa Chen, Wei-Lung Chen, Chien-Cheng Huang, Jui-Yuan Chung

**Affiliations:** a Department of Emergency Medicine, Cathay General Hospital; b Department of Emergency Medicine, Chi-Mei Medical Center; c Department of Biotechnology, Southern Taiwan University of Science and Technology; d Fu Jen Catholic University School of Medicine; e Department of Environmental and Occupational Health, College of Medicine, National Cheng Kung University; f Department of Senior Services, Southern Taiwan University of Science and Technology, Tainan, Taiwan.

**Keywords:** death, emergency department, geriatric, influenza, mortality, prediction

## Abstract

The quick sequential organ failure assessment (qSOFA) score is widely used to assess the risk of sepsis and predict in-hospital mortality in patients with suspected infection. However, its ability to predict mortality among geriatric patients with influenza in the emergency department (ED) remains unclear. Therefore, this study was conducted to delineate this issue.

A retrospective case–control study was conducted on geriatric patients (age ≥65 years) with influenza who visited the ED of a medical center between January 01, 2010, and December 31, 2015. Demographic data, vital signs, past histories, influenza subtypes, and treatment outcomes were included in the analysis. We assessed the accuracy of the qSOFA score in predicting 30-day mortality via logistic regression. Covariate adjustment of the area under the receiver operating characteristic curve (AUROC) via regression modeling was performed too.

In total, 409 geriatric ED patients with mean age of 79.5 years and nearly equal sex ratio were recruited. The mean qSOFA score was 0.55 ± 0.7. The Hosmer–Lemeshow goodness-of-fit test was 0.79 for qSOFA score. Patients with qSOFA score of ≥2 (odds ratio, 4.21; 95% confidence interval [CI], 1.56–11.40) had increased in-hospital mortality. qSOFA score of ≥2 also had excellent in-hospital mortality discrimination with an adjusted AUROC of 0.81 (95% CI, 0.71–0.90). A qSOFA of ≥2 had prominent specificity of 0.89 (95% CI, 0.86–0.92).

An increase in qSOFA score of 2 greatly predicts mortality in geriatric patients with influenza.

## 1 Introduction

Influenza and the aging population are both crucial worldwide issues that may result in considerable morbidity and mortality every year.^[1]^ During the influenza epidemic season, the emergency department (ED) would be flooded with patients complaining of fever, seeking medical assistance. Hospitalization is often needed when associated with pulmonary, cardiovascular, and less frequently, neuromuscular disease complications.^[2]^ A total of 142,000 hospitalizations were estimated to be related to influenza infection,^[3]^ and 40% of them consisted of elderly people (aged ≥65 years).^[4]^ Mortality and complications caused by influenza were noted in >90% of geriatric patients.^[3]^ Therefore, an effective clinical tool is needed to discriminate the severity of influenza infection in geriatric patients, in order to avoid fatal consequences and promote proper utilization of medical resources.

The quick sequential organ failure assessment (qSOFA) score was proposed by the members of the Third International Consensus Definitions for Sepsis and Septic Shock (Sepsis-3) in 2016 for a new definition of sepsis and is frequently used to evaluate the risk of sepsis and predict in-hospital mortality. This scoring system is derived from several simple vital signs, including the mental status, respiratory rate, and blood pressure,^[5]^ which are immediately and easily acquired in the ED setting. It does not require laboratory tests and can be assessed repeatedly to predict poor prognosis in adult patients with sepsis.

Other than identifying the risk of sepsis, the role of the qSOFA score in predicting mortality in geriatric patients with influenza remains unclear. After conducting a keywords search, that is, “death,” “geriatric,” “influenza,” “mortality,” “prediction,” and “qSOFA score” in PubMed and Google Scholar, studies associated on this issue were not found. Therefore, this study was conducted to delineate the role of qSOFA score in predicting mortality in geriatric patients with influenza.

## 2 Methods

### 2.1 Study design, setting, and participants

This study was conducted in an 800-bed university-affiliated medical center. The center has a 40-bed ED with board-certified emergency physicians providing care for approximately 55,000 patients per year.^[6]^ Elderly patients account for about 33% of the total annual number of visiting patients.^[7,8]^ Geriatric patients (aged ≥65 years) visiting the ED between January 1, 2010, and December 31, 2015, would be included if they met the following criteria:

(1) Tympanic temperature (TM) of ≥37.2°C or a baseline TM elevated by ≥1.3°C.^[8,9]^
(2) Influenza infection defined as positive influenza pharyngeal or throat swab test.^[9]^

### 2.2 Variable and primary outcome definition

The qSOFA score was calculated based on the following parameters: systolic blood pressure ≤100 mm Hg, respiratory rate ≥22 breaths per minute, and Glasgow Coma Scale score of <15.^[5]^ Patients who survived at least 30 days were considered “survivors” for this analysis.^[10,11]^ Telephone follow-up was conducted to ascertain 30-day survival if the patient was discharged before 30 days.

### 2.3 Data collection and case and control group assignments

Data regarding the geriatric ED patients who fit the criteria of influenza infection were collected via a retrospective chart review; 479 geriatric ED patients met the criteria of influenza infection, and patients’ information including demographic characteristics, vital signs, past histories, laboratory data, influenza subtype, admission, and 30-day mortality were obtained by an emergency physician. After excluding 70 patients with insufficient data, who were lost follow-up, and transferred patients who had been treated in other hospitals, 409 patients were eventually recruited. Required data that is not recorded in the patient's medical chart was considered negative, and the patient will be eventually excluded. The included patients were then divided into 2 groups, which are the survival and mortality groups, based on their 30-day outcome. All of the variables were used to compare the 2 groups (Table 1).

**Table 1:**
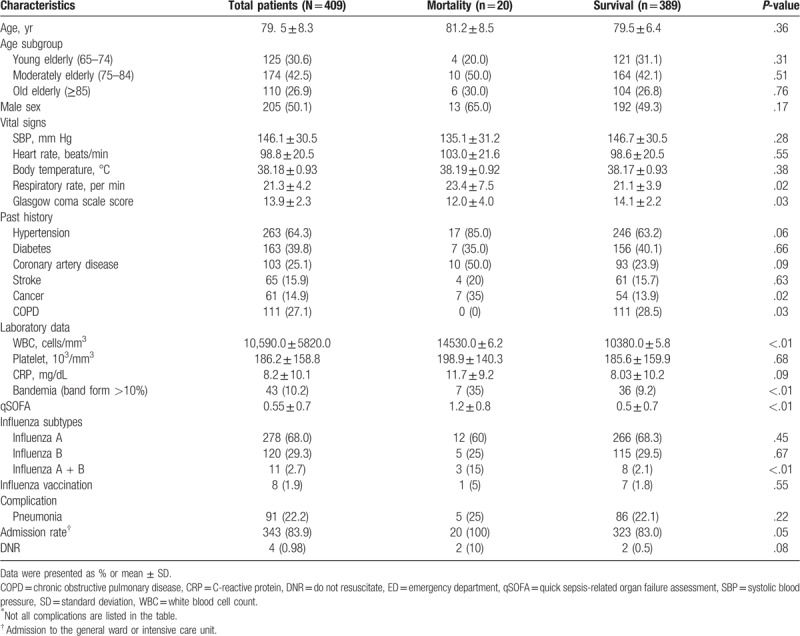
Characteristics of geriatric patients with influenza in the emergency department.

### 2.4 Ethical statement

This study was approved by the Institutional Review Board of the Cathay General Hospital and conducted according to the tenets of the Declaration of Helsinki. Because this was an observational study, the need for patients’ informed consent was waived.

### 2.5 Statistical analysis

SPSS 23.0 for Mac (IBM Corp, Chicago, IL) was used for all statistical analyses. The power was 0.82 using G-power 3.0 for analysis. Continuous data are presented as means ± standard deviation (SD). In the univariate analyses, an independent samples *t* test or the Mann–Whitney–Wilcoxon test was used for continuous variables. Pearson chi-squared test or Fisher exact test was used for categorical variables. Logistic regression was performed to evaluate mortality prediction for qSOFA score of ≥1, ≥2, and ≥3. The most significant (*P* < .05) score range was considered positive. The area under the receiver operating characteristic curve (AUROC) was used to evaluate the mortality discrimination ability of the qSOFA score. The AUROC was further adjusted for by comorbidities that affect mortality (*P* value < .1) via regression modeling. The Hosmer–Lemeshow goodness-of-fit test was performed to evaluate the reliability of the scoring systems. Sensitivity, specificity, positive predictive value, and negative predictive value were also evaluated to determine the performance of positive qSOFA score.

## 3 Results

A total of 409 patients were recruited in this study, with a 30-day mortality rate of 4.9% (20/409) (Table 1). The mean age ± SD was 79.5 ± 8.3, and the percentages of 2 sexes were nearly equal. The mean ± SD of the systolic blood pressure, respiratory rate, and Glasgow Coma Scale score were 146.1 ± 30.5 (mm Hg), 21.3 ± 4.2 (per minute), and 13.9 ± 2.3, respectively. The mortality group had a higher prevalence of cancer than the survival group, but the prevalence of chronic obstructive pulmonary disease was lower. None of the patients signed “do-not-resuscitate” agreement upon admission. During the laboratory data analysis, the mortality group had a higher white blood cell count, percentage of bandemia, and coinfection rate (ie, influenza A + B) than the survival group.

All of the patients were treated with either Oseltamivir or Zanamivir immediately within 24 hours after being diagnosed with influenza infection. Causes of mortality among 20 patients were sepsis (70%, 14 patients), respiratory failure (15%, 3 patients), and cardiovascular event (15%, 2 patients with acute myocardial infarction and 1 with myocarditis). No statistical difference in “do not resuscitate” orders were observed between the mortality and survival groups.

**Figure 1:**
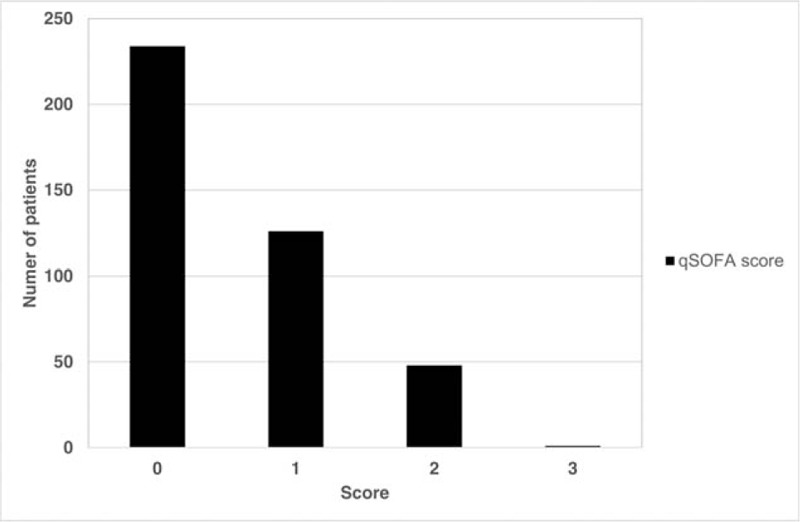
Distribution of patients by qSOFA score among geriatric patients with influenza infection. qSOFA = quick sequential organ failure assessment.

**Figure 2:**
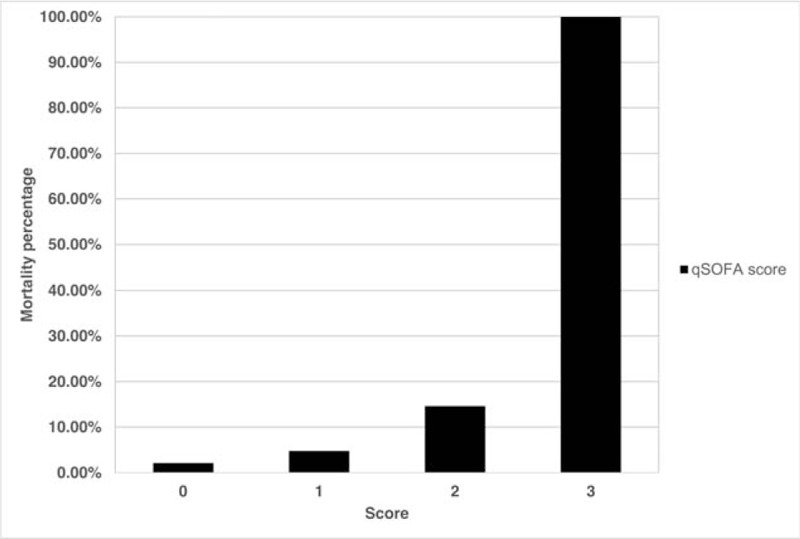
Mortality by qSOFA score among geriatric patients with influenza infection. qSOFA = quick sequential organ failure assessment.

The distribution of geriatric patients with influenza infection according to the qSOFA score showed that 234 (57.2%) patients had 0 point, 126 (30.8%) had 1, and 48 (11.7%) had 2 (Fig. 1). The mortality rate was 14.6% for 2 points and 100% for 3 points according to the qSOFA score (Fig. 2). Logistic regression was performed to predict mortality among patients with qSOFA scores of ≥1, ≥2, and ≥ 3. The most significant qSOFA score was ≥2 (odds ratio, 4.21; 95% confidence interval [CI], 1.56–11.40). The Hosmer–Lemeshow goodness-of-fit was 0.79 for the qSOFA score of ≥2 (Table 2).

**Table 2:**
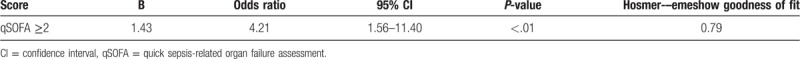
Mortality rate prediction using qSOFA score of ≥2, identified by logistic regression.

The AUROC was adjusted by coronary artery disease (CAD) (*P* = .09) and cancer (*P* = .02). The adjusted AUROC for mortality prediction showed that the qSOFA score of ≥2 (0.81; 95% CI, 0.71–0.90) had an excellent mortality discrimination ability (Table 3). The performance of qSOFA score of ≥2 in predicting mortality in geriatric patients with influenza infection showed a low sensitivity of 0.40 (95% CI, 0.20–0.64) and high specificity of 0.89 (95% CI, 0.86–0.92) (Table 4).

**Table 3:**
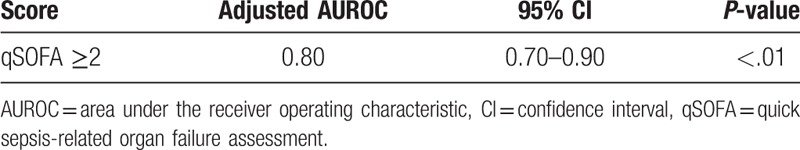
Adjusted AUROC for mortality discrimination of qSOFA score ≥2 in geriatric patients with influenza infection.

**Table 4:**
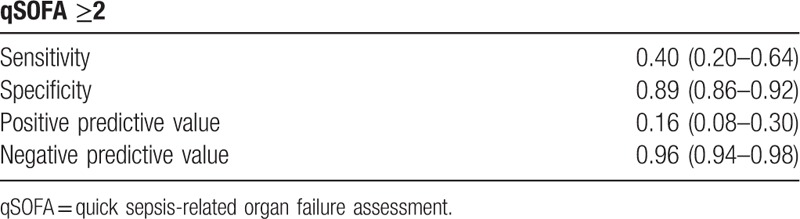
Performance of qSOFA score in predicting mortality in geriatric patients with influenza infection.

## 4 Discussion

The qSOFA score is a useful tool in predicting mortality and organ failure outside the intensive care units in patients with sepsis.^[12]^ In this study, increased qSOFA score of ≥2 also demonstrated good accuracy of mortality prediction in geriatric patients with influenza infection. Although no similar previous study was found, several articles discussed and debated the usefulness of qSOFA score in predicting the prognosis of adults in the ED. A recent multicenter prospective cohort study conducted by Freund et al showed that the qSOFA score is better than SIRS criteria in predicting in-hospital mortality with an AUROC of 0.80 in 879 patients with suspected infection treated at the ED.^[13]^ Williams et al also demonstrated consistent results by analyzing a large Australian cohort of 8871 ED admitted patients with suspected infection, concluding that the qSOFA score of ≥2 is an excellent mortality and organ dysfunction prediction tool.^[14]^

The qSOFA score of ≥2 is highly specific in predicting mortality of geriatric patients with influenza infection. However, fatal outcome was also noted in influenza-infected elderly patients with qSOFA score of <2, as atypical infection presentations, such as hypoxemia, renal failure, coagulopathy, or hyperbilirubinemia, cannot be assessed by the qSOFA score.^[12]^ In this study, despite a lack of statistical significance, 2.5% of elderly patients infected with influenza died with a qSOFA of score <2. Fortunately, the low sensitivity disadvantage of the qSOFA score can be limited by the high number of patients visiting the ED, with low expected mortality rate.^[15]^ Therefore, the specificity seemed to be more important in the ED to avoid unnecessary medical treatment and expenses.

The AUROC was adjusted according to comorbidities, CAD, and cancer that will affect mortality.^[16]^ Past CAD history, combined with influenza infection, may lead to exacerbation of preexisting cardiac disease, deterioration of heart failure, and occurrence of myocardial infarction, leading to increased risk of death.^[17]^ Meanwhile, patients with cancer history may be receiving chemotherapy or radiation therapy, resulting in immunocompromised status, which is vulnerable to infection.^[18]^ The high prevalence of chronic obstructive pulmonary disease (COPD) in this study, compared to the overall geriatric population, was probably due to the impaired pulmonary function of the elderly patients with COPD, which makes them more susceptible to influenza infection.^[19]^

Although influenza vaccination plays an important role in attempts to reduce the mortality burden of influenza, the mortality benefits of influenza vaccination in elderly people is still controversial. Several studies, since 1980, were unable to proof significant decrease in influenza-related mortality, even as vaccination coverage increased from 15% to 65%. In addition, the effectiveness of the influenza vaccine also varies from season to season.^[20]^ Further researches are needed to clarify this issue.

The major strength of this study is that it is the first to report the role of qSOFA score in predicting mortality in geriatric patients with influenza. This study also has some limitations. First, some valuable information and data may be missing due to the retrospective nature of the study. Second, the severity of influenza may be higher as this study was conducted at a medical center. Third, the included elderly patients with influenza infection for >5 years was relatively low. This may be related to the selection of positive influenza swab test as the inclusion criteria rather than the clinical diagnosis. Patients, who were clinically diagnosed with influenza, but with false-negative influenza swab result, may be overlooked, and not included in the study. The influenza swab test has a modest sensitivity of 58% to 67% and a superior specificity of 98%.^[21]^ Fourth, advanced examinations, such as reverse transcriptase-polymerase chain reaction, immunofluorescence assay, or viral culture, should be conducted to further confirm the diagnosis of influenza, as the influenza swab test may have false-positive or negative results. The swab test had a positive predictive rate of 85.7% and negative predictive rate of 89.8% for influenza A and positive predictive rate of 66.7% and negative predictive rate of 93.9% for influenza B.^[22]^ However, the criteria used in this study have the advantage of being simple and practical for the study. Fifth, influenza is a seasonal illness, the variation of influenza virus strains between seasons is crucial as there may consequence in different severities in specific populations, such as elderly people. Therefore, further studies about this issue are warranted.

## 5 Conclusions

The qSOFA score is an effective tool to predict mortality in geriatric ED patients with influenza infection. Geriatric ED patients with an increased qSOFA score of ≥2 had fourfold risk of 30-day mortality than those with qSOFA score of <2. However, the low sensitivity disadvantage of the qSOFA score should be carefully considered; therefore, patients with qSOFA score of <2 should be reassessed and reevaluated. Further studies are needed to validate the findings of this study.

## Author contributions

SHC, CCY, YAC, JYC, CC Hsu, and CC Huang designed and conceived this study and wrote the manuscript. JYC performed the statistical analysis. JHC and WLC provided professional suggestions and wrote the manuscript. All authors read and approved the final manuscript.

**Data curation:** Jui-Yuan Chung.

**Formal analysis:** Jui-Yuan Chung.

**Supervision:** Jiann-Hwa Chen, Wei-Lung Chen.

**Writing – original draft:** Su-Hen Chang, Chien-Chun Yeh, Yen-An Chen, Chien-Chin Hsu, Jui-Yuan Chung.

**Writing – review and editing:** Jiann-Hwa Chen, Wei-Lung Chen, Chien-Cheng Huang, Jui-Yuan Chung.
